# Correlation between Stroke Risk and Systolic Blood Pressure in Patients over 50 Years with Uncontrolled Hypertension: Results from the SYSTUP-India Study

**DOI:** 10.1155/2021/6622651

**Published:** 2021-06-28

**Authors:** Rishi Sethi, J. S. Hiremath, V. Ganesh, Sunip Banerjee, Mahesh Shah, Ashwani Mehta, Preeti Nikam, Minakshi Jaiswal, Nishita Shah

**Affiliations:** ^1^Department of Cardiology, KG's Medical University, Lucknow, India; ^2^Cath Lab, Ruby Hall Clinic, Pune, India; ^3^Vignesh Heart and Diabetes Centre, Annai Arul Hospital, Chennai, India; ^4^S.G. Cardiac Care, Kolkata, India; ^5^Nanavati Hospital, Hinduja Hospital, Breach Candy Hospital, Arogyanidhi, BSES MG Hospital, Mumbai, Maharashtra, India; ^6^Sir Ganga Ram Hospital, New Delhi, India; ^7^Serdia Pharmaceuticals (India) Pvt. Ltd., Mumbai, Maharashtra, India

## Abstract

**Objectives:**

To assess mean systolic and diastolic blood pressure (SBP and DBP) levels in patients ≥50 years with uncontrolled hypertension (HTN) and evaluate the correlation between BP and stroke risk. It also assessed therapeutic drug classes prescribed in these patients.

**Methods:**

A cross-sectional, observational study was conducted at 176 outpatient centers across India, including patients aged ≥50 years with elevated SBP (≥140 mmHg). The relationship between stroke risk, calculated using Stroke Riskometer™, and mean SBP, mean DBP, and other risk factors was evaluated using Pearson correlation coefficient and logistic regression analysis.

**Results:**

The study included 3791 patients (men, 60.0%; mean age: 62.1 ± 8.3 years; mean BMI: 27 kg/m^2^) with mean SBP 157.3 ± 12.8 mmHg and mean DBP 89.8 ± 9.7 mmHg. Five-year stroke risk in 33.9% and 10-year stroke risk in 70% patients were moderate to severe. A ~4% increase in both 5- and 10-year stroke risk with each 1 mmHg increase in mean SBP (*p* < 0.0001) was seen. However, mean DBP did not exhibit any significant correlation with 5-year (*p* = 0.242) or 10-year (*p* = 0.8038) stroke risk. There was a positive correlation between mean SBP and patient age, comorbid diabetes, and smoking and alcohol habits (*p* < 0.0001). Comorbid diabetes and smoking increased 5- and 10-year stroke risk by 2- to 5-fold. Irrespective of the risk category, most patients received antihypertensive therapy with an angiotensin receptor blocker.

**Conclusion:**

Findings corroborate an association between stroke risk and mean SBP. These real-world clinical findings indicate that efforts are required to improve primary prevention of stroke and reduce the prevalence of recurrent stroke in India.

## 1. Introduction

Hypertension (HTN) is a major risk factor for coronary heart disease and cerebrovascular disease and the leading cardiovascular (CV) risk factor for deaths worldwide [1, 2]. The global prevalence of HTN, estimated to be 20,526 per 100,000 persons in 2015, is on a steady rise [3]. The increase in the number of individuals with HTN is associated with a substantial increase in deaths and disability [3]. The number of people with uncontrolled HTN has risen from 594 million in 1975 to nearly 1.13 billion in 2015 [4]. Furthermore, out of the approximately 1.4 billion hypertensive patients globally, only about one in seven achieves adequate blood pressure control [5]. The prevalence of hypertension is reported to be 33.8% in India, with control rates being as low as 7.9% [6, 7]. Moreover, hypertension remains undetected in about 50% otherwise healthy population, as reported by studies in India and China [8, 9].

Undetected and uncontrolled HTN is a major contributor to stroke worldwide [10]. In India, HTN accounts for 57% of all stroke deaths and 24% of all coronary heart disease (CHD) deaths [11]. More than 70% of all strokes occurring each year are first occurrences, and therefore, the primary prevention of stroke is of immense public health importance [12]. In evidence-based guidelines for the management of risk factors to prevent the first occurrence of stroke, the major focus is on the management of modifiable risk factors, including HTN [13].

Aging induces the progressive stiffening of the large arteries and increases wave reflections with a consequent rise in the systolic blood pressure (SBP) and a decrease in the diastolic blood pressure (DBP) [14]. Increased stiffness and the consequent elevation in blood pressure are associated with an increased risk of stroke [15]. In an aging population, elevated SBP is responsible for most cases of uncontrolled HTN [16]. The increased prevalence of elevated SBP with increasing age has been shown to correlate with an increase in stroke mortality rate [17]. The absolute risk of stroke in a patient with a SBP of 140 mmHg is already three times that of a patient with an optimal SBP of 120 mmHg [17]. Therefore, clinical practice guidelines define normal SBP as <120 mmHg [18, 19]. Furthermore, it is well established that SBP lowering is effective for the primary prevention of stroke and recurrent stroke prevention [20, 21].

Identifying populations or individuals that are at high risk for stroke may aid in optimizing stroke prevention initiatives. An accurate and widely accessible stroke risk prediction tool could help clinicians identify patients at high risk and guide patients to appropriately manage their risk factors in a timely manner. The Stroke Riskometer™, developed by the National Institute for Stroke and Applied Neurosciences, Auckland University of Technology, uses an algorithm derived from the Framingham Stroke Risk Score (FSRS) prediction algorithm [22] and includes several major risk factors for stroke (both ischemic and hemorrhagic strokes), largely based on the INTERSTROKE study [23]. The application is endorsed by the World Stroke Organization, World Federation of Neurology, and International Association on Neurology and Epidemiology and provides estimates of the absolute risk of stroke within the next 5 to 10 years for individuals aged between 20 and 90 years [24]. The Stroke Riskometer™ has been successfully validated as being comparable in performance for stroke prediction with the FSRS and QStroke, two widely used stroke risk scoring systems [24].

Renin-angiotensin receptor inhibitors, calcium channel blockers (CCBs), and diuretics (particularly thiazide-like diuretics) are the commonly recommended classes of antihypertensive medications [18, 19, 25]. The selection of appropriate antihypertensive agents for patients is crucial for hypertension management. Moreover, measures to improve adherence, such as the use of fixed-dose combination drugs, also increase the BP control rate and reduce the risk of stroke [13, 26].

The SYSTolic blood pressUre in older Patients with hypertension (SYSTUP), in relation to stroke risk and current antihypertensive treatment strategies, was a cross-sectional, observational study designed to assess mean SBP in patients with uncontrolled HTN and evaluate the correlation between blood pressure and stroke risk in a real-world clinical setting in India. Further, information on different classes of antihypertensive drugs prescribed to hypertensive patients was collected.

## 2. Materials and Methods

### 2.1. Study Design and Data Collection

The SYSTUP study was a single-visit, prospective, noninterventional, observational study conducted at 176 outpatient centers across India. Over a period of 4 weeks, participating physicians each recruited 25 consecutive patients who met the inclusion criteria. Demographic and clinical data, including age, sex, ethnicity, BMI, blood pressure, diabetes, smoking and alcohol habits, family history of and prior CV disease, and physical activity (at least 2.5 hours per week), were collected during the study visit using a structured form. Five-year and 10-year stroke risk score was obtained for each patient using the Stroke Riskometer™ application, which could be downloaded from Google Play (for Android) or App Store (for Apple) [27]. Patients were categorized into four risk categories based on their stroke risk score: low risk, <0.1; moderate risk, 0.1–<0.2; high risk, 0.2–<0.3; and very high risk, ≥0.3. The study was approved by local institutional review boards, and all patients provided informed consent in accordance with national and international guidelines.

### 2.2. Patients

This study included hypertensive patients who presented at the outpatient clinic for a routine consultation. The key inclusion criteria were at least 50 years of age with a confirmed diagnosis of hypertension and elevated SBP, defined as ≥140 mmHg, during the study visit. Blood pressure was measured according to the European Society of Hypertension (ESH) guidelines using an auscultatory or oscillometric semiautomatic sphygmomanometer [28]. Patients remained seated for 3-5 minutes before the BP was measured. The higher of at least two measurements taken 1-2 minutes apart was documented. Patients with any condition that could prevent participation, such as inability to complete the consent form, were excluded.

### 2.3. Statistical Analysis

Categorical parameters were presented using descriptive statistics, and continuous variables were presented as mean ± SD. The relationship between mean SBP/mean DBP and stroke risk was evaluated using the Pearson correlation coefficient. Logistic regression analysis was used to assess the relationship between 5-year and 10-year risk and risk factors, including age, presence of diabetes, physical activity, and smoking and alcohol habits. All analyses were performed using SAS software (version 9.4).

## 3. Results

### 3.1. Patient Characteristics

The analysis included 3791 patients (men, 60.0%). The mean age was 62.1 ± 8.3 years, and the mean BMI is 27.0 ± 8.3 kg/m^2^ (Table 1). Majority of the patients were less than 70 years old (79.4%) and overweight or obese (80.4%), smoked (32.2%), and consumed alcohol (21.6%). Approximately 50% of the patients engaged in regular physical activity. Majority of the patients did not have vascular complications, although diabetes, a known CV risk factor, was present in ~50% of the patients.

### 3.2. Mean Blood Pressure Level and Its Correlation with Age and Stroke Risk

Mean SBP and DBP were 157.3 ± 12.8 mmHg and 89.8 ± 9.7 mmHg, respectively (Table 1). There was a positive correlation between mean SBP and patient age (*r*^2^, 0.172; *p* < 0.0001); mean SBP was higher in older age groups (Table 2). The greatest reduction in stroke risk could be obtained by lowering the BP in the age group ≤ 79 years.

Majority of the patients had a low 5-year stroke risk (66.1%). However, the proportion of patients with low 10-year stroke risk was 30%, while the remaining 70% of patients had moderate to very high risk. A significant positive correlation was found between mean SBP and 5-year (likelihood estimate, 0.038; *p* < 0.0001) and 10-year stroke risk (likelihood estimate, 0.036; *p* < 0.0001) (Table 2). There was an approximate 4% increase in both 5-year and 10-year stroke risk with every 1 mmHg increase in mean SBP. On the other hand, mean DBP did not exhibit any significant correlation with either 5-year (*r*^2^, 0.019; *p* = 0.242) or 10-year stroke risk (*r*^2^, 0.004; *p* = 0.8038) (Table 3).

### 3.3. Correlation between Stroke Risk and Other Risk Factors

Among the risk factors assessed, increasing age, presence of diabetes, smoking, and alcohol consumption (>1 standard drink per day) were all significantly correlated with an increased stroke risk (*p* < 0.0001 for all), while regular physical activity had a significant negative association with stroke risk (*p* < 0.0001) (Table 3). Five-year stroke risk was ~2-fold higher among diabetic patients than those without diabetes (odds ratio (OR), 1.94) and among smokers than nonsmokers (OR, 2.09). Smoking had the strongest impact on 10-year stroke risk (OR, 5.714), followed by alcohol consumption (OR, 3.274) and the presence of diabetes (OR, 2.516).

### 3.4. Stroke Risk and Antihypertensive Medication

**Figure 1:**
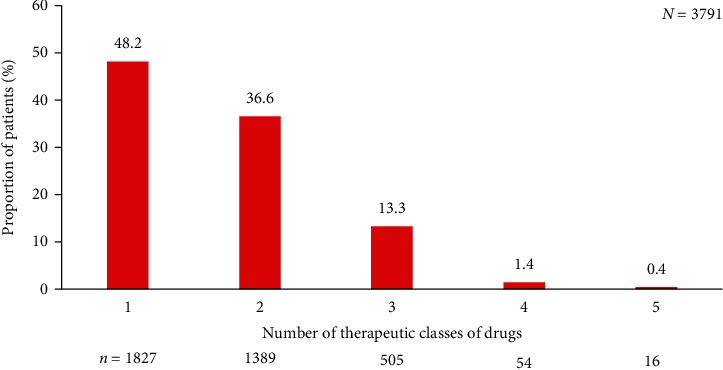
Proportion of patients using one or more classes of antihypertensive drugs.

All patients were receiving antihypertensive therapy. Nearly one-third of the patients (1026/3791) were using a fixed-dose combination (Table 1), and ~15% (575/3791) of the patients were receiving three or more classes of antihypertensive drugs (Figure 1). Commonly prescribed antihypertensive drugs were ARBs (39.2%), CCBs (33.7%), and diuretics (28.4%), while alpha blockers and centrally acting agents were rarely prescribed in these patients (1.4%). The proportion of patients using different classes of antihypertensive drugs did not differ across stroke-risk categories (Table 4).

## 4. Discussion

In this observational study, SBP was found to have a significant impact on both 5-year and 10-year stroke risk in patients over 50 years of age with uncontrolled HTN. Five-year stroke risk was moderate to severe in one-third of the patients, whereas the corresponding proportion of patients for 10-year stroke risk was 70%.

There was a significant positive correlation between age and mean SBP (*p* < 0.0001). These results are in line with observations from other epidemiological studies, which have shown that while DBP increases until the sixth decade of life and decreases thereafter, SBP continues to increase with age [29, 30]. Higher SBP in older age is correlated with an increased CV and cerebrovascular disease risk [29, 30]. Further, it is known that age and HTN are key risk factors for stroke. Hence, we sought to investigate stroke risk among older hypertensive patients.

In addition to age and elevated SBP, lifestyle factors such as obesity, lack of exercise, smoking, alcohol, and stress have been associated with an increased risk of stroke [31]. The relative incidence of stroke compared with myocardial infarction is reported to be significantly higher in women ≥ 65 years and in men > 75 years than younger women and men [32]. This highlights the importance of stroke prevention in patients above the age of 65 years. Lifestyle modifications including weight reduction, low-risk diet, regular physical activity, smoking cessation, and low-to-moderate alcohol consumption may reduce stroke risk by up to 50% [33]. In the present study, risk factors such as increasing age, presence of diabetes, smoking, and alcohol consumption were all associated with higher stroke risk, while regular physical activity was associated with lower stroke risk. These results are in line with previous reports and corroborate the need to actively manage lifestyle factors in older hypertensive patients [30].

Five-year stroke risk was generally low; however, 10-year stroke risk was moderate to very high in a majority of the patients. There were significant positive correlations between mean SBP and 5-year and 10-year stroke risk (*p* < 0.0001). These results underscore the importance of reducing blood pressure in older patients for stroke prevention. It must be noted that all patients included in this analysis were receiving antihypertensive drugs, and 51.7% of the patients were using a combination of two or more classes of antihypertensive drugs (Figure 1). A similar pattern of antihypertensive use was also observed in an international i-SEARCH registry: approximately 31.2% of the patients were receiving 1; 39.7%, 2; and 29.1%, ≥3 drugs [34]. Even though more than half of the patients were treated with combination therapy, the BP control rates were poor in our study (39.2%) and the i-SEARCH registry (21.2%). Low blood pressure control rate could be attributed to inappropriate selection of antihypertensive agents or low adherence to prescribed medications. The most commonly prescribed blood pressure-lowering agents were ARBs followed by CCBs and diuretics, irrespective of stroke risk. Systematic reviews and meta-analyses of trials involving ARBs have shown that ARBs are inferior to angiotensin-converting enzyme (ACE) inhibitors in preventing myocardial infarction and mortality [35]. Moreover, in the 2012 Kidney Disease: Improving Global Outcomes (KDIGO) guideline, ACE inhibitors are stated as the commonly recommended antihypertensive drugs in comorbid hypertension and renal impairment; ARBs are recommended when ACE inhibitors are not tolerated or contraindicated [36]. Moreover, the latest ESC-EASD 2019 guidelines also recommend ACE inhibitors as first-line antihypertensive, and ARBs are recommended only in ACE-intolerant patients [25].

Increasing adherence to treatment has been shown to improve blood control rates and reduce the stroke risk by about one-third [13]. However, majority of hypertension guidelines acknowledge that improving adherence to antihypertensive treatment remains a major challenge for clinical practice in the future [18, 19]. Fixed-dose single-pill combinations (SPCs) have been shown to increase compliance by approximately 26% [26]. In this regard, fixed-dose combinations of perindopril/indapamide and perindopril/indapamide/amlodipine are the only SPCs that are based on ACE inhibitors. The treatment with fixed-dose combinations of perindopril and indapamide and amlodipine has been demonstrated to improve the BP control in several randomized trials and real-world studies [37, 38]. The decrease in BP was associated with a reduction in the risk of nonfatal myocardial infarction, all-cause mortality, total cardiovascular events and procedures, and cardiovascular mortality [39, 40]. Therefore, the use of fixed-dose SPCs could provide a tool to improve the medication adherence and BP control rate in the routine clinical practice in India.

The main limitations of our study were as follows: cross-sectional design; no assessment of adherence to medications, duration of hypertension, gender effect, fatal or nonfatal stroke; and the lack of a detailed analysis of resistant HTN and stroke risk among patients with resistant HTN. However, the key strength of our study is the collection of real-world data across India.

## 5. Conclusion

Overall, in a real-world clinical setting in India, SBP was found to have a significant impact on both 5-year and 10-year stroke risk in patients over 50 years of age with uncontrolled HTN. Majority of the patients were at moderate to very high 10-year stroke risk. In addition to age and elevated SBP, factors such as the presence of diabetes, lack of regular physical activity, and smoking and alcohol habits were all associated with an increased stroke risk. ARBs were the most commonly prescribed antihypertensive agents for older hypertensive patients in clinical practice in India. These findings support the strengthening of primary care to improve primary prevention and reduce the occurrence of stroke.

## Figures and Tables

**Table 1:** Demographic and clinical characteristics of patients.

Characteristic	*N* = 3791
Mean age ± SD (years)	62.1 ± 8.3
Male	2275 (60.0)
Indian ethnicity	3746 (98.8)
Mean BMI ± SD (kg/m^2^)	27.0 ± 8.3
BMI categories	
Underweight	55 (1.5)
Normal	688 (18.1)
Overweight	657 (17.3)
Obese	2391 (63.1)
Smoker	1220 (32.2)
Consumed alcohol (>1 standard drink per day)	819 (21.6)
Consumed enough fruits/vegetables	1990 (52.5)
Physical activity (for at least 2.5 hours per week)	1941 (51.2)
Mental/emotional stress	1345 (35.5)
Family history of stroke/heart attack < 65 y	918 (24.2)
Diabetes	1922 (50.7)
Peripheral artery disease	847 (22.3)
Left ventricular hypertrophy	572 (15.1)
Atrial fibrillation	281 (7.4)
Cognitive problem	342 (9.0)
Poor memory	727 (19.2)
Brain injury	89 (2.3)
Previous stroke or TIA	313 (8.3)
Mean SBP	157.3 ± 12.8
Mean DBP	89.8 ± 9.7
Uncontrolled mean DBP	2304 (60.8)
Antihypertensive treatment	3791 (100)
Fixed-dose combination therapy	1026 (27.1)

Values represent *n* (%) unless specified otherwise. BMI: body mass index; TIA: transient ischemic attack.

**Table 2:** Correlation between mean systolic BP and age, 5-year stroke risk, and 10-year stroke risk.

	*n*	MSBP	Correlation	*p* value
Age				
50-59 years	1623	155.61 ± 12.45	0.172^a^	<0.0001
60-69 years	1387	157.13 ± 12.00		
70-79 years	634	159.99 ± 14.13		
>80 years	147	164.86 ± 14.39		
5-year stroke risk				
Low (<0.1)	2504	155.12 ± 11.69	−6.72 (0.44)^b^	<0.0001
Moderate (0.1–<0.2)	769	160.41 ± 14.20		
High (0.2–<0.3)	255	161.75 ± 13.75		
Very high (≥0.3)	263	164.07 ± 12.93		
10-year stroke risk				
Low (<0.1)	1119	153.50 ± 11.36	−4.76 (0.50)^b^	<0.0001
Moderate (0.1–<0.2)	1176	155.88 ± 11.68		
High (0.2–<0.3)	583	160.36 ± 13.29		
Very high (≥0.3)	913	161.66 ± 13.92		

^a^Data represent Pearson correlation coefficient (*r*^2^). ^b^Data represent maximum likelihood estimate for intercept (standard error).

**Table 3:** Correlation between CV risk factors and stroke risk.

		5-year stroke risk					10-year stroke risk				
	*N*	Low	Moderate	High	Very high	OR/*r*^2^	Low	Moderate	High	Very high	OR/*r*^2^
Total	3791	2504 (66.1)	769 (20.3)	255 (6.7)	263 (6.9)	—	1119 (29.5)	1176 (31.0)	583 (15.4)	913 (24.1)	—
Age											
50–59 years	1623	1315 (34.7)	231 (6.1)	49 (1.3)	28 (0.7)	—/0.415^∗∗^	840 (22.2)	433 (11.4)	170 (4.5)	180 (4.7)	—/0.453^∗∗^
60–69 years	1387	892 (23.5)	280 (7.4)	116 (3.1)	99 (2.6)		248 (6.5)	557 (14.7)	219 (5.8)	363 (9.6)	
70–79 years	634	268 (7.1)	197 (5.2)	76 (2.0)	93 (2.5)		27 (0.7)	173 (4.6)	154 (4.1)	280 (7.4)	
>80 years	147	29 (0.8)	61 (1.17)	14 (0.4)	43 (1.2)		4 (0.1)	13 (0.3)	40 (1)	90 (2.4)	
BMI category											
Underweight	55	36 (0.9)	11 (0.3)	2 (0.1)	6 (0.2)	—/−0.030	14 (0.4)	17 (0.4)	8 (0.2)	16 (0.4)	—/−0.038^∗^
Normal	688	430 (11.3)	150 (4.0)	45 (1.2)	63 (1.7)		192 (5.1)	200 (5.3)	107 (2.8)	189 (5.0)	
Overweight	657	458 (12.1)	124 (3.3)	42 (1.1)	33 (0.9)		187 (4.9)	238 (6.3)	95 (2.5)	137 (3.6)	
Obese	2391	1580 (41.7)	484 (12.8)	166 (4.4)	161 (4.2)		726 (19.2)	721 (19.0)	373 (9.8)	571 (15.1)	
Diastolic BP^a^											
Uncontrolled	2304	1487 (39.2)	494 (13.0)	158 (4.2)	165 (4.4)	—/0.019	626 (16.5)	729 (19.2)	371 (9.8)	578 (15.2)	—/0.004
Controlled	1487	1017 (26.8)	275 (7.3)	97 (2.6)	98 (2.6)		493 (13)	447 (11.8)	212 (5.6)	335 (8.8)	
Diabetes											
Yes	1922	1130 (29.8)	442 (11.7)	160 (4.2)	190 (5.0)	1.943/0.665^∗∗^	392 (10.3)	616 (16.2)	321 (8.5)	593 (15.6)	2.516/0.923^∗∗^
No	1869	1374 (36.2)	327 (8.6)	95 (2.5)	73 (1.9)		727 (19.2)	560 (14.8)	262 (6.9)	320 (8.4)	
Physical activity											
Yes	1941	1340 (35.3)	354 (9.3)	122 (3.2)	125 (3.3)	0.759/−0.276^∗∗^	613 (16.2)	624 (16.5)	264 (7.0)	440 (11.6)	0.829/0.187^∗∗^
No	1850	1164 (30.7)	415 (10.9)	133 (3.5)	138 (3.6)		506 (13.3)	552 (14.6)	319 (8.4)	473 (12.5)	
Smoker											
Yes	1220	666 (17.6)	306 (8.1)	115 (3.0)	133 (3.5)	2.09/0.739^∗∗^	122 (3.2)	449 (11.8)	226 (6.0)	423 (11.2)	5.714/1.743^∗∗^
No	2571	1838 (48.5)	463 (12.2)	140 (3.7)	130 (3.4)		997 (26.3)	727 (19.2)	357 (9.4)	490 (12.9)	
Alcohol											
Yes	819	459 (12.1)	191 (5.0)	84 (2.2)	85 (2.2)	1.733/0.550^∗∗^	112 (3.0)	278 (7.3)	154 (4.1)	275 (7.3)	3.274/1.186^∗∗^
No	2972	2045 (53.9)	578 (15.2)	171 (4.5)	178 (4.7)		1007 (26.6)	898 (23.7)	429 (11.3)	638 (16.8)	

**Table 4:** Therapeutic class of drugs stratified by risk of stroke.

Therapeutic class		5-year stroke risk (*N* = 3791)				10-year stroke risk (*N* = 3791)			
	Total	Low	Moderate	High	Very high	Low	Moderate	High	Very high
*N*	**3791**	**2504**	**769**	**255**	**263**	**1119**	**1176**	**583**	**913**
ACE inhibitor	658 (17.4)	424 (16.9)	118 (15.3)	62 (24.3)	54 (20.5)	154 (13.8)	224 (19.0)	99 (17.0)	181 (19.8)
Angiotensin receptor blocker	1487 (39.2)	916 (36.6)	337 (43.8)	123 (48.2)	111 (42.2)	412 (36.8)	427 (36.3)	242 (41.5)	406 (44.5)
Beta blocker	840 (22.2)	507 (20.2)	198 (25.7)	59 (23.1)	76 (28.9)	212 (18.9)	239 (20.3)	144 (24.7)	245 (26.8)
Calcium channel blocker	1276 (33.7)	845 (33.7)	256 (33.3)	87 (34.1)	88 (33.5)	371 (33.2)	402 (34.2)	207 (35.5)	296 (32.4)
Diuretic	1077 (28.4)	643 (25.7)	241 (31.3)	98 (38.4)	95 (36.1)	275 (24.6)	320 (27.2)	159 (27.3)	323 (35.4)
Alpha blocker or CAA	52 (1.4)	28 (1.1)	14 (1.8)	4 (1.6)	6 (2.3)	11 (1.0)	15 (1.3)	9 (1.5)	17 (1.9)

Values represent *n* (%). *N* represents the total number of patients and the denominator for calculating the percentages in each column. The total of percentages in any column is higher than 100 as some patients were receiving more than one antihypertensive drug and hence included each drug class. ACE: angiotensin-converting enzyme; CAA: centrally acting agent.

## Data Availability

The datasets used and analyzed during the current study are available from the corresponding author on reasonable request.
